# Predicting the efficacy of donepezil intervention in Alzheimer’s disease patients using regional homogeneity in the inferior orbitofrontal cortex

**DOI:** 10.1007/s40520-023-02691-6

**Published:** 2024-04-17

**Authors:** Min Dai, Zhongwei Guo, Honglian Xia, Hong Zhu, Jiapeng Li, Hongtao Hou, Guizhi Zhao, Xiaozheng Liu

**Affiliations:** 1 Department of Radiology, The Second Affiliated Hospital and Yuying Children’s Hospital, Wenzhou Medical University, 109 Xueyuan North Road, Wenzhou, 325027 Zhejiang China; 2 Tongde Hospital of Zhejiang Province, 234 Gucui Road, Hangzhou, 310012 Zhejiang China; 3 Wenzhou Key Laboratory of Structural and Functional Imaging, Wenzhou, China

**Keywords:** Alzheimer's disease, Depression, Functional magnetic resonance imaging, Donepezil, Regional homogeneity

## Abstract

**Background:**

Although donepezil is a commonly used drug for treating Alzheimer's disease (AD), the mechanisms by which it affects patients’ functional brain activity, and thus modulates clinical symptoms, remain unclear.

**Methods:**

In the present study, we used resting-state functional magnetic resonance imaging (MRI) and regional homogeneity (ReHo) to investigate the effects of donepezil on local brain activity in AD patients. Resting-state functional MRI data were collected from 32 subjects: 16 healthy controls and 16 AD patients. All 16 AD patients underwent 6 months of donepezil treatment and received two MRI scans (pre- and post-intervention). Analysis of covariance and post hoc analyses were used to compare ReHo differences among the healthy controls, pre-intervention AD patients, and post-intervention AD patients. Pearson correlation analysis was used to examine relationships between ReHo values in differential brain regions and clinical symptoms.

**Results:**

Compared with healthy controls, post-intervention AD patients had reduced ReHo in the orbital part of the inferior frontal gyrus, and pre-intervention AD patients had reduced ReHo in the orbital part of the right inferior frontal gyrus. Pattern recognition models revealed that pre-intervention ReHo values in abnormal brain regions of AD patients were 76% accurate for predicting the efficacy of donepezil on cognitive function and 65% accurate for predicting its efficacy on depressive symptoms.

**Conclusions:**

These findings deepen our understanding of the brain mechanisms underlying the clinical efficacy of donepezil in AD patients, and provide a novel way to predict its clinical efficacy in such patients.

## Introduction

Donepezil hydrochloride is currently the first-line drug for treating Alzheimer's disease (AD) and is widely used in the clinic because of its effectiveness against cognitive dysfunction, low side effects, good compliance, and high efficacy and acceptability [1]. Donepezil may improve cognitive function in patients with AD by inhibiting the activity of acetylcholinesterase [2], which is involved in cell development and maturation and can promote neuronal development and nerve regeneration [3]. However, the mechanisms by which donepezil modulates brain function in AD patients, and thus interferes with clinical symptoms, remain unclear.

In recent years, functional magnetic resonance imaging (fMRI) has been used to study the mechanisms of donepezil intervention in the AD brain [4–7]. An MRI-based structural imaging study revealed that donepezil slows the process of forebrain atrophy in AD patients compared with placebo [4]. In contrast, after donepezil intervention, patients with mild cognitive impairment have increased putamen, globus pallidus, and inferior frontal gyrus (IFG) gray matter volume, but decreased hippocampal volume [5]. A resting-state fMRI (rsfMRI) study demonstrated increased orbitofrontal network connectivity in AD patients after a 12-week intervention with donepezil; orbitofrontal network connectivity was associated with cognitive improvement [6]. Regional homogeneity (ReHo) has also been used to study the mechanisms of donepezil intervention in the AD brain. Cheng et al. [7] explored changes in ReHo after donepezil intervention in 11 AD patients, and reported decreased ReHo in the right gyrus rectus, right precentral gyrus, and left superior temporal gyrus after the intervention. However, their analysis did not correct for multiple comparisons and the sample size was very small.

The aim of the present study was to use rsfMRI to examine alterations in ReHo in AD patients after donepezil intervention compared with healthy controls (HCs). Based on previous studies of the brains of AD patients before and after donepezil-based interventions [4–6], we hypothesized that, relative to baseline status and the HC group, the post-intervention AD group would display altered ReHo in regions of the orbitofrontal gyrus (OFG) that are critical to cognitive and emotional regulation [4–6].

## Materials and methods

### Participants

Sixteen subjects with mild or moderate AD completed this longitudinal study from January 2018 to July 2022 at Hangzhou, Zhejiang, China. Subjects with AD met probable AD criteria according to the National Institute on Aging-Alzheimer's Association guidelines [8]. Subjects were right-handed with a Clinical Dementia Rating score of 0.5, Mini-Mental State Examination (MMSE) score < 24, and > 6 years of education. Sixteen healthy subjects were recruited as HCs at Tongde Hospital. They had no cognitive impairment and a Clinical Dementia Rating score of 0. Participants were excluded if they had the following conditions: a history of psychiatric disorders, were taking antidepressant drugs, or had MRI contraindications. All participants signed informed consent forms. The study was approved by the local Ethics Committee (2017-11-12).

Severity of depression was rated using the Cornell Scale for Depression in Dementia [9] and the Neuropsychiatric Inventory (NPI) [10]. Depressive symptoms were considered present when the Cornell Scale for Depression in Dementia score was ≤ 6 and the NPI depression domain score was ≥ 4 [9–11].

### MRI scanning

A 3.0 Tesla MRI scanner with an eight-channel head coil (Siemens Magnetom Verio, Siemens Medical Systems, Erlangen, Germany) was used. Axial functional images were obtained using a gradient echo-planar imaging sequence with the following parameters: repetition time (TR) = 2000 ms, echo time (TE) = 30 ms, slices = 33, thickness = 4.8 mm, gap = 0 mm, field of view (FOV) = 200 mm × 200 mm, acquisition matrix = 64 × 64, and flip angle (FA) = 90°. The fMRI process took 6 min and 40 s. High-resolution three-dimensional magnetization-prepared rapid gradient-echo T1-weighted imaging sequences were obtained using the following parameters: inversion time (TI) = 900 ms, TR = 1900 ms, TE = 2.48 ms, slices = 128, thickness = 1 mm, gap = 0 mm, FOV = 256 mm × 256 mm, acquisition matrix = 256 × 256, and FA = 9°.

### Data processing

SPM12 (http://www.fil.ion.ucl.ac.uk/spm) and Resting-State fMRI Data Analysis Toolkit plus V1.25 (RESTplus V1.25) (http://www.restfmri.net) were used for data preprocessing. The first ten volumes were removed, slice-timing was performed, and motion correction was applied. Participants had low head motion in terms of framewise displacement (mean < 0.5). We initially coregistered fMRI pictures to each person's high-resolution T1 anatomical scan and then normalized them to the MNI152 template before they were normalized to Montreal Neurological Institute space. Next, we performed linear detrending and temporal band-pass filtering (0.01–0.08 Hz). Cerebrospinal fluid, white matter, and head motion artifacts were regressed out of each voxel's time series to eliminate spurious signals.

### ReHo calculations

The preprocessed images were then used to perform ReHo computation. Individual ReHo maps were created using Kendall’s coefficient of concordance to determine the degree of resemblance between a given voxel's time series and that of its 26 closest neighbors. The ReHo maps were then normalized by dividing the value of each voxel by the global average. Finally, a Gaussian kernel (full width at half maximum = 6 mm) was used to spatially smooth the standardized mean ReHo maps.

### Statistical analysis

Statistical software was used to analyze demographic and clinical traits (Statistical Package for the Social Sciences v.15.0; SPSS, Inc., Chicago, IL, USA). Age and education differences were assessed using a two-sample *t* test, and sex distribution differences were assessed using the Chi-squared test.

The ReHo maps were compared voxel-by-voxel among the three groups using one-way analysis of covariance (ANCOVA). We then extracted brain masks that displayed significant differences in the ANCOVA analysis. Finally, we performed post hoc *t* tests between each pair of groups using the ANCOVA brain masks. The two-sample *t* test was used for the post hoc comparison of the AD and HC groups, and the paired *t* test was used to compare the pre- and post-intervention AD groups. We removed the mean relative head motion displacements of age and sex as covariates in the ANCOVA and *t* tests to ensure the accuracy of the results. With an individual voxel *p* value < 0.005 and a cluster size > 25 voxels, the significance level was set at *p* < 0.05 (AlphaSim-corrected for multiple comparisons). We used the REST Slice Viewer (http://www.restfmri.net) to display the differential brain regions and the Automated Anatomical Labeling 3 standard template was used as the reference standard for brain region localisation.

### Relationship between ReHo values and clinical variables

We extracted the mean ReHo values of abnormal brain regions and used Pearson correlation analysis to investigate the relationships between abnormal ReHo values and clinical variables in AD patients pre- and post-intervention (*p* < 0.05).

### ReHo-based efficacy prediction

We developed pattern recognition models to evaluate the ability of ReHo values to predict the efficacy of donepezil intervention in AD. Pre-intervention ReHo values in AD abnormal brain regions were used as input variables and post-intervention efficacy measures were used as output variables. Post-intervention efficacy was divided into two categories: cognitive function (assessed using the MMSE) and depressive symptoms (assessed using the NPI). When comparing MMSE scores pre- and post-intervention, ΔMMSE > 0 was considered good cognitive function efficacy. For NPI scores, a 50% increase was considered good depressive symptom efficacy. We built the prediction model using the fitcnb and fitcknn functions of MATLAB 2019b with a fivefold cross-check. We then calculated the area under the curve to evaluate the accuracy of ReHo values for predicting intervention efficacy.

## Results

### Neuropsychological results

Demographic and clinical data are shown in Table 1. There were no significant between-group differences in age, sex distribution, or educational attainment (*p* = 0.107, 0.157, and 0.240, respectively). Compared with HCs, the AD group had significantly lower MMSE scores and higher NPI scores (*p* < 0.001).

**Table 1:** Demographic and neuropsychological data

	AD group	HCs group	c2/t-value	*p *value
Gender, *n* (M/F)	16 (8/8)	16(7/9)	2	0.157
Age, years	65.2 ± 8.1	69.1 ± 4.5	1.659	0.107
Education, years	9.1 ± 2.0	8.3 ± 2.1	1.198	0.240
Duration, months	15 ± 8.8			
MMSE	19.7 ± 2.6	29.1 ± 0.90	-10.342	< 0.001
MMSE(24w)	20.1 ± 2.5			
NPI	4.56 ± 2.9	0.15 ± 0.36	12.632	< 0.001
NPI(24w)	1.38 ± 1.2			
CSDD	3.25 ± 2.40			
CSDD(24w)	0.75 ± 0.71			

Data are presented as the mean ± standard deviation. The Chi-squared test was used to compare sex, and two-sample *t* tests were used to compare age and neuropsychological data

*HCs* healthy controls, *AD* Alzheimer’s disease, *M* male, *F* female, *MMSE* Mini-Mental State Examination, *NPI* neuropsychiatric inventory, *CSDD* Cornell scale for depression in dementia

### Abnormal ReHo values in the AD group

**Fig. 1:**
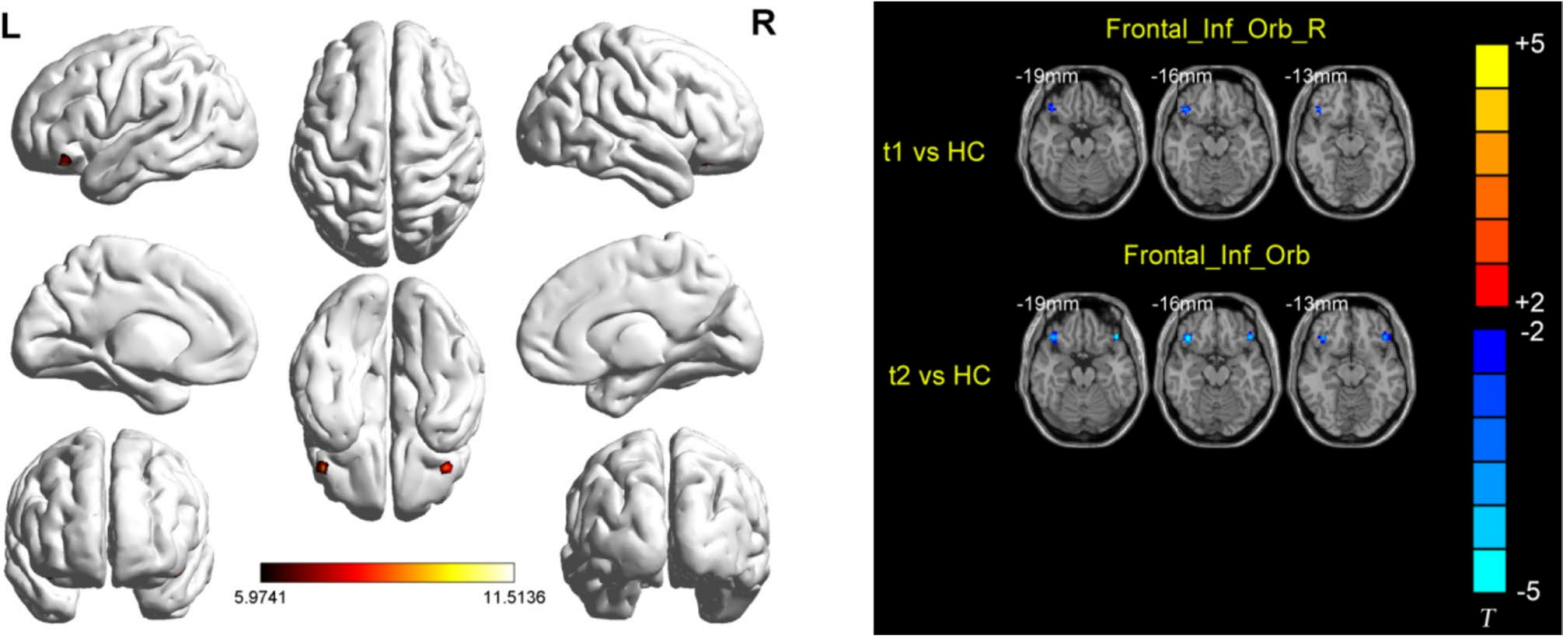
Brain regions showing abnormal regional homogeneity values among the three groups. Left figure: Differential brain regions obtained from three groups of ANCOVA analysis; Right figure: Brain regions showing significantly different regional homogeneity values in the post-intervention Alzheimer’s disease (AD) group compared with the pre-intervention AD group and the healthy control group: t1, pre-intervention AD group; t2, post-intervention AD group

Between-group analysis revealed altered ReHo in the pre- and post-intervention AD groups relative to the HC group across multiple regions (Fig. 1, Table 2). Compared with HCs, post-intervention AD patients had reduced ReHo in the inferior frontal gyrus/orbit part (IFG.orb) (Fig. 1, Table 2), and pre-intervention AD patients had reduced ReHo in the right IFG.orb (Fig. 1, Table 2).

**Table 2:** Brain regions with significantly different regional homogeneity values in the post-intervention AD group compared with the pre-intervention AD group and the HC group

Brain regions	Voxels	BA	MNI coordinates			*F/T* value	*P* value
			x	y	z		
ANCOVA							
Frontal_Inf_Orb_R	121	47	42	33	− 15	11.513	< 0.01
Frontal_Inf_Orb_L	67	47	− 45	33	− 18	11.317	< 0.01
Frontal_Sup_L	77	10	− 3	57	11	9.0134	< 0.01
T1 vs HCs							
Frontal_Inf_Orb_R	91	47	57	36	− 3	− 4.6966	< 0.02
T2 vs HCs							
Frontal_Inf_Orb_R	100	47	39	30	− 15	− 4.1881	< 0.01
Frontal_Inf_Orb_L	56	47	− 45	33	− 18	− 4.2388	< 0.01

*AD* Alzheimer’s disease, *ANOVA* analysis of variance, *MNI* Montreal Neurological Institute, *BA* Brodmann area, *HC* healthy control, *t1* pre-intervention AD group, *t2* post-intervention AD group

### Correlations between ReHo and clinical variables

**Fig. 2:**
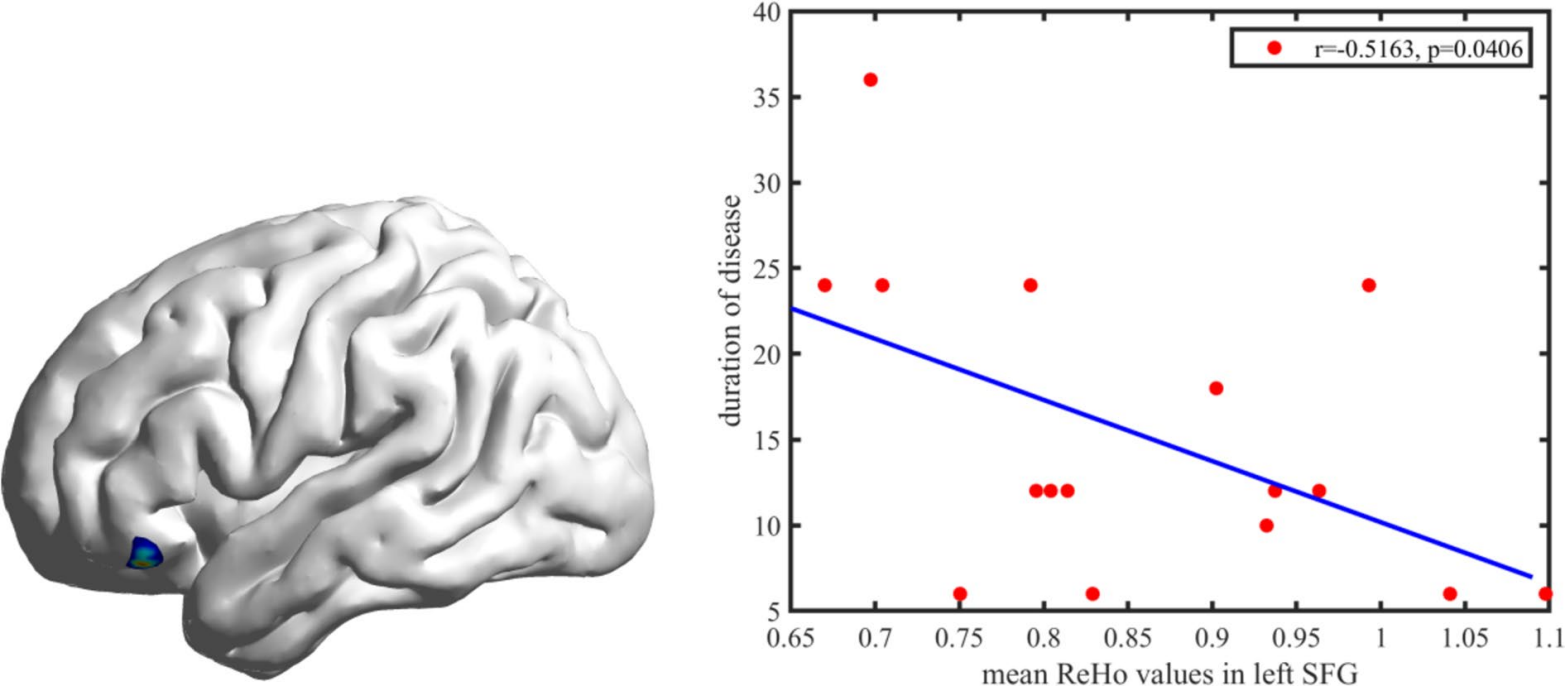
Pearson correlation analysis showing a significant negative correlation between regional homogeneity values of the left superior frontal gyrus (SFG) and duration of disease in post-intervention Alzheimer’s disease patients

There was a significant correlation between ReHo values in the left superior frontal gyrus and duration of disease in post-intervention AD patients (*r* =  − 0.5163, *p* = 0.0406) (Fig. 2).

### Predictive performance of ReHo values

**Fig. 3:**
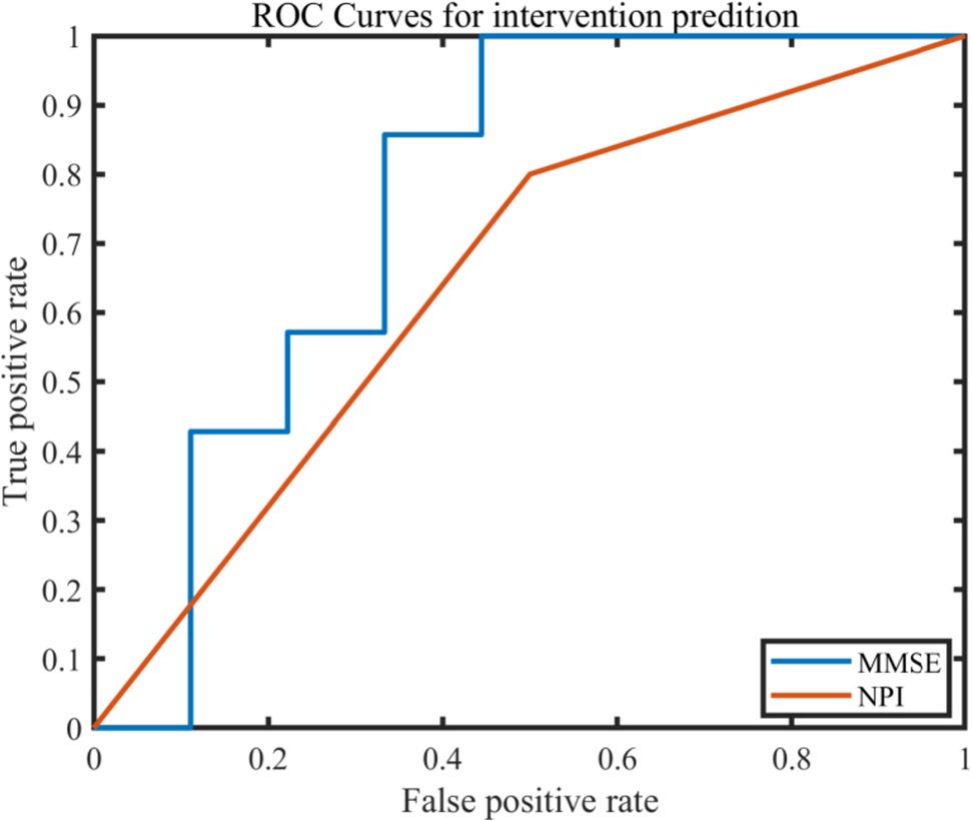
Receiver-operating characteristic plot of regional homogeneity values for predicting intervention efficacy. The predicted area under the curve (AUC) value for Mini-Mental State Examination improvement after intervention was 0.7619. The predicted AUC value for Neuropsychiatric Inventory improvement after intervention was 0.6500

A naïve Bayes model showed that pre-intervention ReHo values of abnormal brain regions in AD patients were 76% accurate for predicting the efficacy of the intervention on cognitive function. A k-nearest neighbor classification showed that pre-intervention ReHo values of abnormal brain regions in AD patients were 65% accurate for predicting the efficacy of the intervention on depressive symptoms (Fig. 3).

## Discussion

In the present study, we used rsfMRI data to examine ReHo in AD patients both before and after donepezil intervention relative to HCs. Compared with HCs, AD patients had reduced ReHo in the IFG.orb. Moreover, pre-intervention ReHo values of abnormal brain regions in AD patients were 76% accurate for predicting the efficacy of donepezil intervention on cognitive function and 65% accurate for predicting its efficacy on depressive symptoms.

The results of the ReHo analysis revealed that IFG.orb was the main abnormal brain region between the three groups. Neurofibrillary tangle pathology is widespread in the orbital prefrontal cortex of AD patients [12]. The OFG receives and integrates input from multiple senses, such as vision, taste, and smell [13]. In particular, olfactory system dysfunction is closely linked to AD [14, 15]. Sedghizadeh et al. collected olfactory data from AD patients and HCs and used high-frequency frontal oscillations in response to olfactory stimuli to classify AD and HCs with an accuracy of 91.7% [16]. Mega et al. [17] divided 30 AD patients into three groups based on behavioral responses to donepezil (responders, non-responders, and no change). Compared with non-responders, responders had more significant de-inhibitory and euphoric sensations as well as significantly lower OFG and dorsolateral frontal perfusion. This suggests that orbitofrontal syndrome predicts behavioral responses to cholinesterase inhibitor treatment in AD patients.

The OFG is a major part of the limbic system [18], and a previous study has suggested that the neurobiological mechanisms underlying depression may include abnormal activity in limbic system brain regions [18]. Furthermore, resting-state functional connectivity between the left anterior cingulate cortex and left OFG was reduced in individuals with major depressive disorder and bipolar disorder compared with healthy controls [19]. Matt et al. [20] used ultrasound brain stimulation as a clinical intervention for AD patients and reported post-intervention improvements in both cognitive function and depressive symptoms. In addition, functional connectivity differences between the salient network and left OFG were reduced compared with healthy controls. Our results suggest that the OFG is an important target for intervention in AD patients and is the main imaging marker for predicting the efficacy of donepezil intervention in AD patients.

Some limitations to our study should be noted. First, although fMRI studies have shown that a sample size of 13 subjects per group ensures 80% statistical power, relatively small sample sizes can affect the robustness of statistical results. Second, different imaging modalities allow changes in brain function to be observed from different perspectives, for example, the changes in white matter [21]. Thus, the combination of different imaging methods should be used to explore multimodal imaging markers for predicting the efficacy of donepezil intervention in AD. Third, MRI data were collected after 24 weeks of donepezil intervention only. Collecting MRI data after 4, 8, and 16 weeks of intervention may improve our understanding of brain changes with donepezil intervention in patients with AD.

## Conclusions

In the current study, we compared ReHo among AD patients before and after donepezil intervention and HCs. Abnormal brain regions were located in the OFG. These findings may allow a better understanding of the brain mechanisms of donepezil intervention in AD and the corresponding imaging markers.

## Data Availability

The data are available from the authors upon reasonable request.
